# Exploring associations between positive psychological and school adjustment of EFL learners using the PERMA framework: roles of coping style and negative emotions

**DOI:** 10.3389/fpsyg.2026.1906415

**Published:** 2026-09-28

**Authors:** Cui Jing, Wanmin Zhang

**Affiliations:** Foreign Languages Teaching Department, Hainan Vocational University of Science and Technology, Haikou, Hainan, China

**Keywords:** coping style, EFL learners, negative emotion, positive psychology, school adjustment

## Abstract

**Background:**

Positive psychology has been associated with students’ school adjustment, but the indirect associations underlying this relationship remain underexplored, particularly in EFL learners.

**Methods:**

An online survey was conducted with 462 university students to examine the associations among positive psychology, coping style, negative emotion, and school adjustment. Indirect and sequential indirect associations were examined using PROCESS Model 6 in SPSS.

**Results:**

Positive psychology was positively associated with school adjustment. Significant indirect associations were observed through coping style (effect = 0.025, 95% CI [0.002, 0.052]) and negative emotion (effect = 0.152, 95% CI [0.095, 0.216]), accounting for 3.61 and 21.93% of the total association, respectively. The sequential indirect association involving coping style and negative emotion was also significant (effect = 0.025, 95% CI [0.011, 0.042]), accounting for 3.61%.

**Conclusion:**

These findings highlight the relevance of positive psychological resources, coping style, and emotional experiences to school adjustment among EFL learners. They also provide potential directions for future research examining student support and intervention approaches, while causal or intervention effects cannot be inferred from the present cross-sectional data.

## 1 Introduction

With the acceleration of globalization and the widespread use of internet technologies, English as a Foreign Language (EFL) learners face multiple challenges in the modern educational environment, particularly in adapting to school life. EFL learners not only need to overcome language barriers but also deal with cultural differences, variations in teaching methods, and social adaptation issues. Therefore, school adjustment (SA) has become a crucial factor affecting both their academic success and mental health (Bai et al., 2022). For these learners, effective SA enhances learning efficiency, aids in stress management, and improves social skills (Chen et al., 2022; San and Guo, 2023). Thus, it is of significant practical importance to examine the factors associated with SA among EFL learners.

In educational and psychological studies, student SA has grown in significance. Existing studies have explored its associations with multiple domains of student functioning, such as academic achievement, social relationships, and emotional well-being. Prior research has shown that certain beneficial mental elements, including pleasant feelings, social bonds, and life meaning, co-occur with better adaptation results in student populations (Basharpoor et al., 2022; Xiang, 2024). Additionally, coping style (CS), as a key strategy for individuals to manage stress, has been linked to students’ adjustment-related outcomes (Li et al., 2024; Stanisławski, 2019). By comparison, students reporting elevated levels of negative emotion (NE) tend to exhibit lower levels of psychological adjustment, and NE has been consistently related to indicators of psychological distress and reduced academic functioning (Cao et al., 2023; Chen and Chen, 2020; Chen and Zhang, 2024; Wang et al., 2016).

Although existing research has provided valuable insights into students’ SA, studies have often examined positive psychology (PPs), CS, and NE separately or within different explanatory models (Chen and Guo, 2026; Kao, 2024; Wang et al., 2021). Previous research has documented associations between positive psychological factors and SA (Umucu et al., 2024), between coping strategies and adjustment (Wu et al., 2024), and between negative emotional experiences and students’ academic or psychological functioning (Cao et al., 2023). However, less is known about how CS and NE may be simultaneously associated with the relationship between PPs and SA. In particular, examining CS and NE within the same model may help distinguish the role of coping-related responses from that of emotional experiences in relation to students’ adjustment.

Compared with previous research, the contribution of the present study is not based solely on its focus on EFL learners. First, by incorporating CS and NE into the same analytical model, this study examines their respective indirect associations between PPs and SA and allows their relative magnitudes to be compared. This may provide a more differentiated understanding of whether coping-related processes and negative emotional experiences show similar or different associations with students’ adjustment. Second, the study further examines a theoretically specified sequential pathway involving CS and NE, thereby considering not only their separate indirect associations but also the statistical relationship between coping-related and emotional factors within the proposed model. Third, examining these relationships among EFL learners provides evidence from an educational context in which students may encounter language-learning, academic, and interpersonal adjustment demands. Thus, the EFL context provides a relevant setting for examining the proposed relationships rather than constituting the sole source of the study’s contribution.

The main objective of this research is to examine the associations among PPs, CS, NE, and SA among EFL learners. In particular, the study investigates the association between PPs and SA and examines whether CS and NE are separately and sequentially associated with this relationship. Based on this, the study poses the following research questions:

1. Is PPs significantly associated with EFL learners’ SA?
2. Is there a significant sequential indirect association between PPs and SA through CS and NE?

Through the proposed statistical model, this study further examines the separate indirect associations through CS and NE and the sequential indirect association through CS and NE. The contribution of this approach lies in distinguishing the relative roles of coping-related and emotional factors rather than merely combining several previously studied constructs. Given the cross-sectional design, however, the proposed sequence represents a theoretically specified statistical model and should not be interpreted as establishing temporal or causal relationships among PPs, CS, NE, and SA.

## 2 Literature review and research hypotheses

### 2.1 PPs and the PERMA model

PPs is a branch of psychology that gained prominence through the work of Csikszentmihalyi and Seligman (2000). It focuses on studying human strengths and virtues, aiming to enhance well-being and quality of life. Unlike traditional psychology, which primarily focuses on treating mental illness and psychological problems, PPs emphasizes the promotion of mental health and happiness by strengthening individuals’ positive resources (Csikszentmihalyi and Seligman, 2000). Current research in PPs centers around “one core and three pillars,” with subjective well-being at the core and supported by positive emotional experiences, positive personality traits, and positive social systems. Subjective well-being, the core of PPs, represents the ideal state of human flourishing. According to Seligman (2011), true well-being should not be measured by a single factor but should encompass both subjective and objective components. Based on the concept of flourishing, Seligman (2011) proposed the PERMA model of well-being.

The PERMA model forms a measurable, multidimensional framework in PPs, consisting of five key elements: positive emotions, engagement, relationships, meaning, and accomplishments (Seligman, 2011). These five dimensions are all quantifiable and together focus on achieving the core goal of well-being in PPs. Positive emotions refer to good feelings like happiness, engagement refers to being fully absorbed in an activity, relationships involve close connections with others, meaning refers to a sense of purpose, and accomplishments reflect feelings of success and achievement (Seligman, 2011). The PERMA model, a key framework in PPs, has seen broad application in fields such as education (Lou and Xu, 2022), organizational behavior (Donaldson et al., 2022), and clinical psychology (Fang et al., 2023). PERMA-related dimensions have been associated with students’ motivation (Jie et al., 2022), academic performance (Séllei et al., 2021), and SA (Umucu et al., 2024) by fostering positive emotions, promoting engagement and interaction, emphasizing meaning and supporting the experience of accomplishment.

### 2.2 PPs and SA

Existing research shows a significant correlation between PPs and SA. PPs has been consistently associated with students’ SA in educational settings (Cho and Frizzell, 2023). Specifically, engaged students tend to face academic and social challenges with a positive attitude, which helps them adapt better to the school environment. Moreover, positive emotions have been associated with lower stress experiences and better interpersonal functioning. Chang et al. (2020) proposed that positive emotions play a role in building and sustaining strong social ties, and these ties matter greatly for SA (Shrivastava et al., 2022). A sense of meaning and accomplishment provides students with motivation and goals in both academic and personal life, and relates to students’ perceived resilience in confronting adversity (Choi et al., 2023). For example, Yu et al. (2022) explored the impact of learning engagement based on PPs on EFL learners’ adjustment. The literature emphasizes that cultivating students’ positive psychological dimensions under the PERMA model may provide psychological resources that are associated with better SA among EFL learners. Therefore, positive psychological traits may be particularly relevant to SA among EFL learners, providing a basis for examining their association with students’ adjustment in the present study. Drawing on the above discussion, the following hypotheses are proposed:

*H1*: PPs is significantly positively correlated with EFL learners’ SA.

### 2.3 The indirect role of CS

The PPs dimensions of the PERMA model, such as positive emotions, meaning, and a sense of accomplishment, are significantly associated with the selection and use of CSs (Zou et al., 2024). Findings show that learners with stronger positive psychological resources tend to adopt more adaptive coping strategies. These traits are associated with individuals’ tendency to maintain a positive attitude when facing stress and challenges and to adopt more adaptive coping responses (Wu et al., 2020). Using 376 university students as a sample, Zhang et al. (2024) explored how physical activity, CS, and positive emotions relate to one another, with positive emotions treated as a potential intermediary. Their findings indicated that positive emotions were positively associated with students’ CS.

CS is a key variable that is associated with SA (Sulejmani and Pop-Jordanova, 2026a). Individuals who habitually adopt positive coping strategies tend to use problem-solving approaches rather than emotion-oriented ones (Stanisławski, 2019). These coping patterns have also been associated with a more positive self-concept and more favorable academic and social outcomes (Marsh and Martin, 2011). For example, Wu et al. (2024) conducted a survey with 1,488 Chinese middle school students, reporting that CS was positively related to SA and partially explained the relationship of SA with physical activity. Therefore, this study suggests that positive psychological traits are equally important for EFL learners, especially in addressing challenges in language learning, as positive coping strategies have been associated with more favorable SA outcomes. Based on the above analysis, the study hypothesizes:

*H2*: PPs is indirectly associated with SA through CS.

### 2.4 The indirect role of NE

Positive emotions, meaning, and accomplishment, as core components of the PERMA framework, have frequently been examined in relation to NE. Existing research indicates that these positive psychological characteristics are associated with lower levels and intensity of negative emotions, which may be relevant to how individuals respond to stressful events and challenging tasks (Li et al., 2021; Shi et al., 2023). Zhang et al. (2022) surveyed 739 university students to examine the associations among self-concept, resilience, and NE, with resilience considered as a potential mediator. The findings showed that resilience was negatively associated with NE and showed an indirect association in the relationship between self-concept and NE.

NE has been negatively associated with students’ SA. High levels of NE, such as anxiety, depression, and anger, are associated with poorer academic performance, social skills, and mental health, further affecting their overall level of SA (Chen and Chen, 2020; Wang et al., 2016). Choi and Min (2015) conducted a survey of 319 South Korean high school students, examining the effect of adolescent anxiety on SA. In this study, adolescent anxiety was negatively associated with school adjustment. For EFL learners, NE may also be negatively associated with language-learning outcomes, including learning motivation and classroom participation. Therefore, cultivating PPs may help EFL learners develop psychological resources that are associated with better emotional regulation and SA outcomes. Drawing on the preceding literature, the following hypothesis is advanced:

*H3*: PPs is indirectly associated with SA through NE.

### 2.5 Sequential indirect association through CS and NE

According to emotion regulation theory, when individuals face stressful or challenging events, coping strategies represent cognitive and behavioral responses used to manage perceived demands and have been found to be associated with individuals’ emotional experiences. Students reporting higher levels of CS generally report lower levels of NE, according to studies that show a substantial negative association between positive CS and NE. Liao et al. (2024) examined the association between CS and NE in nursing students, with their results showing a negative correlation between constructive coping methods and NE occurrence. More precisely, adaptive coping strategies may be associated with more effective responses to stress and with lower levels of anxiety and depression (Wang et al., 2020). Problem-solving approaches relate to more active stress handling and reduced helplessness and anxiety, whereas support-seeking relates to less loneliness and depression through emotional and practical aid (Xiong et al., 2019). The proposed ordering of CS before NE is theoretically based on the view that coping reflects how individuals respond to perceived academic or interpersonal demands, whereas NE reflects the emotional experiences associated with those demands and responses. From this perspective, students’ coping responses may be associated with their emotional experiences. This provides a theoretical rationale for specifying CS before NE in the proposed sequential model.

Current research has shown that positive coping strategies are associated with lower levels of NE and more favorable adjustment outcomes (Choi and Min, 2015; Wang et al., 2020). PPs is positively associated with the use of adaptive CS, helping individuals manage stressors and potentially corresponding with lower levels of NE (Liao et al., 2024; Wu et al., 2020). Based on these findings, this study further explores whether the association between PPs and university students’ SA is statistically consistent with a sequential indirect pathway through CS and NE. Nevertheless, the proposed ordering of CS before NE should be regarded as theoretically specified rather than temporally established. With a cross-sectional approach, the reverse sequence where NE comes before CS, along with potential bidirectional links between CS and NE, remains plausible and receives additional attention in a later section. Given the cross-sectional design, the proposed ordering represents a theoretically specified statistical model rather than an empirically established causal sequence. Therefore, the study hypothesizes:

*H4*: PPs is indirectly associated with SA through the sequential pathway of CS and NE.

Figure 1 presents the hypothesized model of the associations among PPs, CS, NE, and SA.

**Figure 1:**
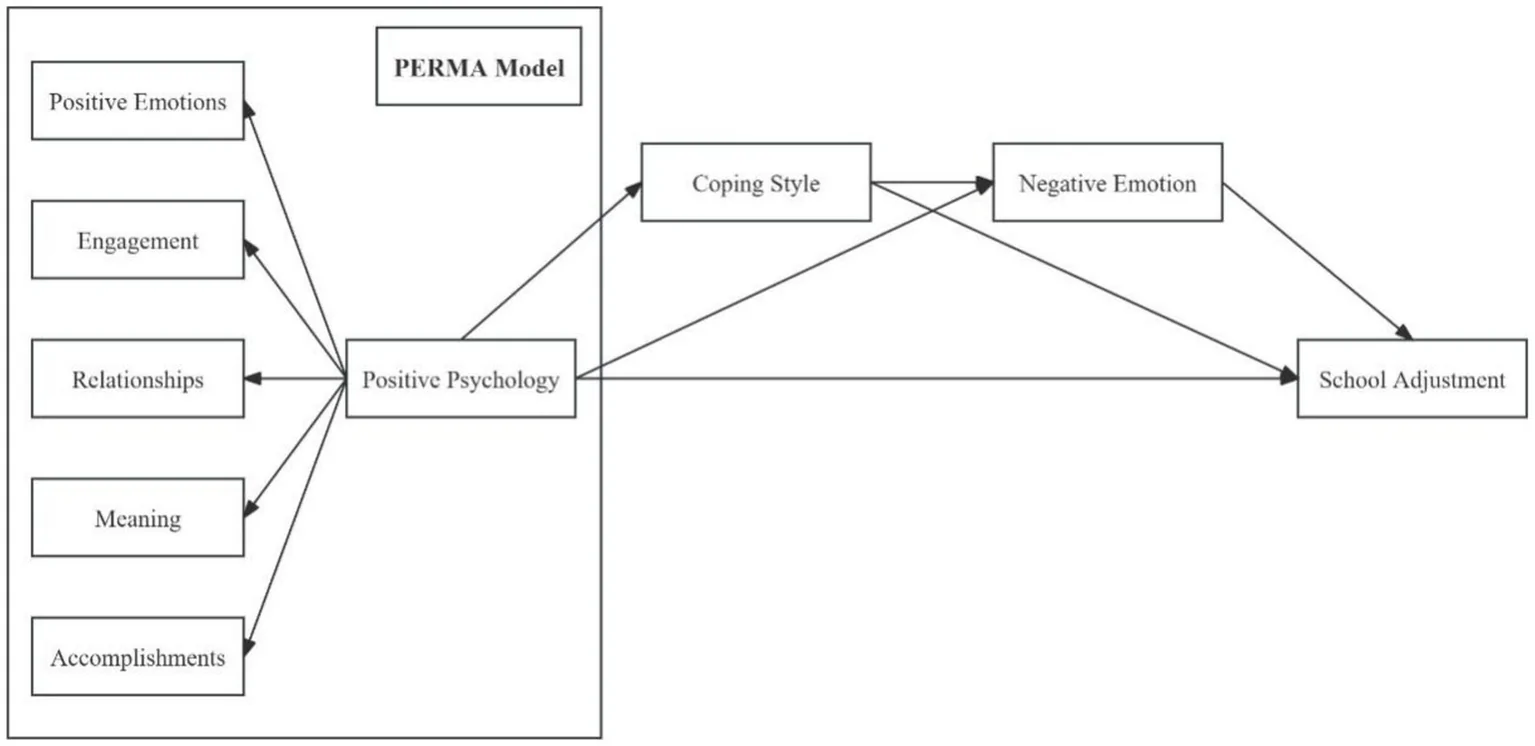
Hypothesized statistical model of the associations among PPs, CS, NE, and SA.

## 3 Materials and methods

### 3.1 Participants

This study employed a cross-sectional research design, utilizing four standardized self-report questionnaires. The participant pool came from one university located in Hainan Province, with recruitment carried out via convenience sampling. Therefore, the sample should not be considered representative of the broader EFL learner population. Data were collected through an online survey on the platform Sojump (www.Sojump.com) to evaluate the hypothesized model. The data collection period lasted from May to June 2026. This study was approved by the Ethics Committee of Hainan Vocational University of Science and Technology (Approval Number: HVUST-2026-12). All participants were informed about the purpose and procedures of the study before participation. Participants aged 18 years or older provided written informed consent themselves. For participants younger than 18 years, written informed consent was obtained from a parent or legal guardian, and assent was obtained from the participants themselves. Participation was voluntary, and participants’ data were kept confidential. The online survey had all questionnaire items configured as required fields. As a result, item-level omissions did not appear in the collected responses. Before participant recruitment and data collection, an *a priori* power analysis was conducted using G*Power 3.1. Assuming a medium effect size (*f*^2^ = 0.15), *α* = 0.05, statistical power of 0.95, and six predictors in the most complex regression model, the analysis indicated a minimum required sample size of 146 participants. A total of 520 students were invited to participate, of whom 496 completed the questionnaire, yielding a response rate of 95.38%. During the screening process, questionnaires were excluded if they had duplicate IP addresses, identical responses for more than 95% of the items, unusually short completion times, or if the respondents were not EFL learners. After screening, 462 valid questionnaires were retained for the final analysis, representing a valid-case retention rate of 93.15%. The final sample therefore exceeded the minimum sample size indicated by the *a priori* power analysis. Table 1 displays the participants’ specific demographic data.

**Table 1:** Demographic information of the sample.

Demographic characteristics	Category	Quantity	Percentage (%)
Gender	Male	219	47.40
	Female	243	52.60
Age	≤18 years old	35	7.58
	19–22 years old	272	58.87
	≥ 23 years old	155	33.55
Grade	Freshman	32	6.93
	Sophomore	121	26.19
	Junior	247	53.46
	Senior	62	13.42

### 3.2 Measurement instruments

The questionnaire consisted of two parts: the first part collected demographic information from the participants, while the second part focused on the study’s constructs. To assess these constructs, well-validated scales were used. The PPs Questionnaire, based on the PERMA model, was adapted from the well-being scale developed by Butler and Kern (2016). It includes 15 items and uses a 7-point Likert scale. For this research, the scale showed strong internal reliability, producing a Cronbach’s *α* value of 0.960. The SA Scale, adapted from the Chinese College Student Adjustment Scale (CCSAS) developed by Fang et al. (2005), focused on SA with 8 items, 3 of which required reverse scoring, and used a 5-point scale, with a Cronbach’s *α* of 0.800. The CS Scale, revised by Zhang Y. Y. et al. (2021), was a simplified version with 6 selected items (3 from each dimension) on a 4-point scale, with a Cronbach’s *α* of 0.885. NEs were measured using the Depression Anxiety Stress Scale (DASS) developed by Lovibond and Lovibond (1995), which included 21 items on a 5-point scale, with a Cronbach’s *α* of 0.965 in this study.

### 3.3 Statistical analysis

Data analyses were conducted using SPSS 22.0. An examination of common method bias (CMB) relied on two distinct techniques, namely Harman’s single-factor test and the common latent factor (CLF) approach. For the study variables, descriptive statistics and Pearson correlation procedures were run to provide data summaries and inspect the interrelationships present. Before the regression analyses, the distributional characteristics of the study variables were examined using skewness and kurtosis. Standardized residual plots were examined for linearity and equal variance. Normal probability plots were used to check the distributional shape of the residuals. Tolerance and variance inflation factor (VIF) figures were used to examine multicollinearity. No major departures from the regression assumptions appeared in the data. Subsequently, the proposed sequential indirect-association model was tested using Model 6 of the PROCESS macro, with gender, age, and grade level included as control variables. The statistical significance of the indirect pathways was determined using bootstrapping with 5,000 resamples and 95% confidence intervals (CIs). All variables were standardized before conducting the PROCESS analyses. Therefore, the regression coefficients and indirect effects reported in the mediation analyses were based on standardized estimates, and the percentages of indirect effects were calculated using standardized indirect effects relative to the standardized total effect.

## 4 Results

### 4.1 Common method Bias test

To evaluate CMB, this study used two approaches. Harman’s single-factor test showed that the largest factor explained 35.806% of the variance, below the 40% benchmark (Podsakoff et al., 2003). Yet the single-factor test by Harman served merely as a preliminary check and does not eliminate the possibility of common method variance.

Additionally, we evaluated CMB using a common latent factor (CLF) analysis. The baseline measurement model showed acceptable model fit (*χ*^2^ = 2346.5, *df* = 1,112, CFI = 0.920, TLI = 0.912, RMSEA = 0.049, SRMR = 0.076), whereas the model including a latent common factor demonstrated comparable fit (*χ*^2^ = 2344.1, *df* = 1,111, CFI = 0.922, TLI = 0.914, RMSEA = 0.049, SRMR = 0.065). The two models showed no significant statistical difference (Δ*χ*^2^ = 2.4, Δ*df* = 1, *p* > 0.05), with the common latent factor yielding little enhancement in model fit. Still, one should not take these results as proof that CMB was absent.

### 4.2 Correlation analysis

The means, standard deviations, skewness, kurtosis, and Pearson correlations for every variable are displayed in Table 2. The data meet the criteria for parametric tests since they are fairly regularly distributed, as indicated by the skewness (Sk) values being all less than 2 and the kurtosis (Kur) values being less than 7. Pearson’s correlation analysis indicated that PPs was positively associated with CS (*r* = 0.268, *p* < 0.001) and SA (*r* = 0.685, *p* < 0.001), and negatively associated with NE (*r* = −0.494, *p* < 0.001). CS was negatively correlated with NE (*r* = −0.164, *p* < 0.001) and positively associated with SA (*r* = 0.172, *p* < 0.001). NE was negatively correlated with SA (*r* = −0.528, *p* < 0.001).

**Table 2:** Means, standard deviations, and correlation analysis of variables (*r*).

Variable	M ± SD	Sk	Kur	PPs	CS	NE	SA
PPs	77.20 ± 16.13	−0.561	0.560	1			
CS	16.10 ± 2.29	0.359	2.730	0.268***	1		
NE	48.28 ± 15.46	0.365	0.347	−0.494***	−0.164***	1	
SA	27.18 ± 4.51	0.251	0.533	0.685***	0.172***	−0.528***	1

*N* = 462. ***p < 0.001, the same below.

### 4.3 Multicollinearity diagnosis

Tolerance and VIF figures served in the evaluation of multicollinearity. Table 3 presents VIFs from 1.232 to 1.586, far under the usual cutoff of 5. Tolerance levels all stayed above 0.1. These values fell within acceptable ranges (Hair et al., 2019). Inspection of the residual diagnostics did not indicate substantial violations of linearity, homoscedasticity, or residual normality.

**Table 3:** Results of multicollinearity diagnostics.

Constructs	Tolerance	VIF
PPs	0.631	1.586
CS	0.812	1.232
NE	0.661	1.512

### 4.4 Analysis of indirect associations

The correlation analysis results indicated significant associations among the study variables, supporting further examination of the indirect associations involving CS and NE. The hypothesized indirect associations were estimated using PROCESS Model 6, with gender, age, and grade level included as covariates (see Tables 4, 5).

**Table 4:** Regression analyses for the proposed sequential model.

Outcome variable	Predictor variable	*R*	R^2^	*F*	*β*	*t*	Bootstrap 95% CI	
							LLCI	ULCI
CS	PPs	0.300	0.090	11.263***	0.291	6.359***	0.201	0.381
NE	PPs	0.585	0.342	47.371***	−0.567	−13.950***	−0.647	−0.487
	CS				−0.313	−7.862***	−0.391	−0.235
SA	PPs	0.746	0.557	95.283***	0.491	12.308***	0.413	0.570
	CS				0.085	2.442*	0.017	0.154
	NE				−0.269	−6.978***	−0.344	−0.193

*β* represents regression coefficients estimated using standardized variables. ***p < 0.001; *p < 0.05.

**Table 5:** Indirect associations through CS and NE.

Effect	Pathways	Effect value	Bootstrap 95% CI		Percentage
			LLCI	ULCI	
Total effect	PPs → SA	0.693	0.578	0.710	100%
Direct effect	PPs → SA	0.491	0.413	0.570	70.85%
Indirect effect	PPs → CS → SA	0.025	0.002	0.052	3.61%
	PPs → NE → SA	0.152	0.095	0.216	21.93%
	PPs → CS → NE → SA	0.025	0.011	0.042	3.61%

All effect estimates are based on standardized variables. Percentages were calculated by dividing each standardized indirect effect by the standardized total effect.

Using a bootstrap technique and the PROCESS macro in SPSS 22.0, the hypothesized indirect associations were examined. Table 5 and Figure 2 display the outcomes from the proposed sequential model. The total effect of PPs on SA was significant (effect = 0.693, 95% CI [0.578, 0.710]), and the direct association between PPs and SA remained significant (effect = 0.491, 95% CI [0.413, 0.570]). Bootstrap analyses further indicated significant indirect associations through CS (effect = 0.025, 95% CI [0.002, 0.052]) and NE (effect = 0.152, 95% CI [0.095, 0.216]). In addition, the sequential indirect association involving CS and NE was significant (effect = 0.025, 95% CI [0.011, 0.042]). The relative proportions of these three indirect associations were 3.61%, 21.93%, and 3.61%, respectively. These percentages represent the relative contribution of each specific indirect pathway and should not be interpreted as cumulative additional effects. The relatively larger proportion attributable to the NE pathway suggests that NE showed a stronger indirect association with the relationship between PPs and SA compared with the other indirect pathways. By contrast, the smaller proportions of the CS pathway and the sequential pathway indicate that these pathways represented relatively smaller but still statistically significant components of the overall association between PPs and SA. All bootstrap confidence intervals excluded zero. Every indirect pathway reached statistical significance. Overall, these results provide statistical support for the hypothesized indirect associations involving CS and NE, including the sequential indirect association, while not implying a causal sequence among these variables.

**Figure 2:**
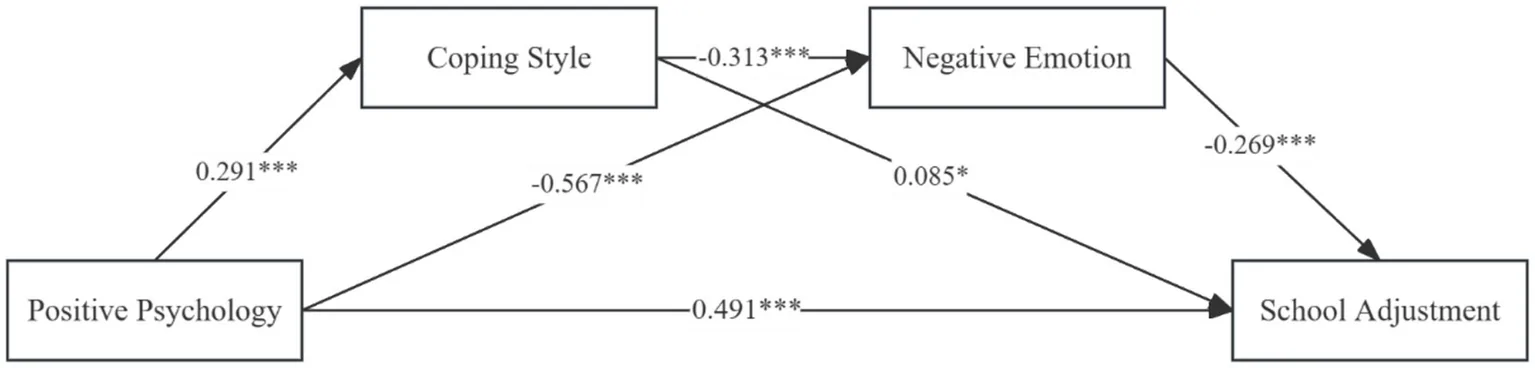
Estimated sequential indirect-association model.

## 5 Discussion

This study, based on the PERMA model, examined associations among PPs, SA, CS, and NE in EFL learners, with particular attention to the potential sequential indirect associations involving CS and NE. Based on prior literature, it was hypothesized that PPs would be positively associated with SA, with CS and NE potentially accounting for part of these statistical associations. The findings were consistent with the proposed statistical model by showing that PPs was positively associated with SA and that CS and NE were involved in these associations. All proposed hypotheses were supported. Yet with a cross-sectional design, these results should be interpreted as statistical associations, not as proof of causal pathways.

PPs was positively associated with SA among EFL learners. This observation corroborates prior findings in the literature (Yu et al., 2022), which reported positive correlations between PPs and students’ learning motivation and school adjustment. Dimensions of PPs, such as positive emotional states, meaning, and accomplishment, were associated with students’ capacity to manage academic and personal challenges and with their level of SA (Zou et al., 2024). These results point to PPs as a possible psychological asset that relates to more positive academic experiences. For EFL learners, who often encounter language-related challenges, performance pressure, and uncertainty during learning processes, positive psychological resources may be particularly relevant for maintaining engagement and adjustment. In addition, students with higher PPs scores tended to report greater engagement in academic activities and campus life, which may correspond with a stronger sense of school belonging. Social relationships, as part of PPs, were also observed to be related to supportive networks, providing emotional resources and contributing to better SA (Zhang X. et al., 2021). By applying the PERMA framework to EFL learners, the present findings extend previous research by illustrating how multiple dimensions of positive psychological functioning may be associated with adjustment experiences in a language-learning context.

The indirect association between PPs and SA through CS was significant but relatively modest (effect = 0.025), accounting for 3.61% of the total association. This finding suggests that coping strategies may represent one possible pathway through which positive psychological resources are associated with school adjustment, but coping behaviors alone may not fully explain how EFL learners adapt to academic demands. This pattern matches earlier work where PPs showed a positive link to CS, and CS in turn showed a link to SA (Wu et al., 2024; Zhang et al., 2024). Recent evidence further suggests that adaptive coping patterns are closely associated with emotional functioning and adjustment outcomes among adolescents and young adults, highlighting the importance of coping processes in psychological adaptation (Sulejmani and Pop-Jordanova, 2026a). Dimensions of PPs, such as positive emotions and engagement, were associated with higher confidence and motivation when facing academic or interpersonal challenges, which corresponded with greater use of adaptive coping strategies rather than avoidance or negative coping methods (Chang et al., 2020). However, the relatively small magnitude of this indirect association indicates that coping strategies may function as a complementary psychological process rather than the primary explanation for the association between PPs and SA in the present model. Therefore, the contribution of CS should be interpreted cautiously and viewed as complementary to other psychological processes involved in school adjustment.

In this sample of EFL learners, NE was significantly associated with both PPs and SA. The indirect association through NE showed a larger effect estimate than the pathway involving CS, indicating that emotional experiences may represent a relatively more proximal correlate of school adjustment in this sample. This finding aligns with previous research suggesting that higher PPs scores are associated with lower NE, which in turn is related to better SA (Choi and Min, 2015; Zhang et al., 2022). Recent studies have further emphasized that psychological distress and negative emotional experiences among young individuals are multidimensional phenomena involving anxiety, stress, and emotional functioning, suggesting that emotional experiences represent an important component of adaptation processes (Sulejmani and Pop-Jordanova, 2026b). One possible explanation is that emotional experiences directly reflect students’ psychological responses to academic and interpersonal environments. For EFL learners, negative emotional experiences such as anxiety, frustration, and stress may be associated with how students perceive their learning experiences, social interactions, and sense of adjustment. Therefore, emotional functioning may constitute an especially important component when examining how positive psychological resources relate to school adjustment. Compared with coping strategies, which mainly reflect cognitive and behavioral responses to challenges, negative emotional experiences may more directly represent students’ immediate psychological states during academic adjustment, which may explain the stronger indirect association observed in the present model. Dimensions of PPs were also associated with stronger emotional regulation abilities, which corresponded with more effective management of NE and adaptive responses to academic and interpersonal challenges (Li et al., 2021; Shi et al., 2023). Also, students with higher PPs tended to report broader and more supportive social networks, which were linked to lower NE and, consequently, higher SA (Chen et al., 2023). Overall, these results point to a broader understanding of the PERMA framework—one that includes not only the existence of positive psychological assets but also their connection to students’ negative affective states.

Among EFL learners, CS and NE were sequentially associated with the relationship between PPs and SA. Although the sequential indirect association was statistically significant, its magnitude was relatively smaller than the indirect association involving NE alone. This pattern suggests that emotional experiences may play a stronger role than coping strategies in accounting for the association between PPs and SA in the present sample. This result echoes earlier reports in which students scoring higher on PPs also reported more adaptive coping, and those coping methods related to fewer NEs and better SA (Liao et al., 2024; Wang et al., 2020; Yu et al., 2022). Theoretically, coping strategies may be related to how students respond to academic and interpersonal demands, whereas NE reflects their emotional experiences during these challenges. Therefore, coping and emotional processes may represent interconnected aspects of adjustment rather than independent processes. However, because CS and NE were measured concurrently, the present findings cannot determine whether coping strategies precede emotional experiences or whether emotional experiences shape subsequent coping responses. Thus, the sequential indirect association should be interpreted as aligning with the theoretical ordering proposed, not as proof of a fixed time-based order. Overall, these findings contribute to the PERMA framework by highlighting that student adjustment may depend not only on positive psychological resources but also on how these resources are associated with emotional experiences and coping responses under academic challenges. This perspective extends traditional PERMA-based interpretations by emphasizing the relevance of negative emotional experiences in understanding student adjustment.

## 6 Implications and limitations

### 6.1 Implications

This study extends the theoretical understanding of PPs in the context of SA among EFL learners. By examining both CS and NE as sequentially associated psychological variables, the study highlights how multiple dimensions of PPs relate to students’ adaptive outcomes. The results show that NE plays a relatively stronger role in the association between PPs and SA compared with CS, suggesting that emotional experiences may be particularly closely associated with students’ adaptation. The findings also support the relevance of the PERMA framework for examining student adjustment. Positive emotions, engagement, meaning, and accomplishment all relate clearly to coping, emotional states, and overall school adaptation. Overall, these findings add to a fuller picture of the psychological factors linked to EFL learners’ school adaptation and broaden the theoretical basis for later work on positive resources and student adjustment.

The findings also provide potential directions for future intervention research and student support practices. However, because the present study employed a cross-sectional observational design, the observed associations cannot demonstrate that interventions targeting PPs, NE, or CS would lead to improvements in SA. Instead, these factors may be considered potential targets for further examination in future intervention studies. In particular, given the relatively larger indirect association involving NE, future research could examine whether interventions aimed at helping EFL learners recognize and manage negative emotional experiences are associated with subsequent improvements in SA. Positive psychology activities targeting positive emotions, engagement, supportive relationships, meaning, and accomplishment may also warrant further evaluation. In addition, adaptive coping strategies, such as problem-solving and support-seeking, could be examined as complementary intervention targets, although the relatively modest indirect association through CS suggests that their role should not be overstated. Longitudinal studies, randomized controlled trials, and other prospective intervention designs are needed to determine whether changes in these psychological factors are followed by improvements in school adjustment.

### 6.2 Limitations and future research directions

Several limitations should be acknowledged when interpreting the findings of this study. First, the cross-sectional nature of this work constrains conclusions about time order and causal direction among PPs, CS, NE, and SA. While grounded in the PERMA framework, the proposed model does not preclude alternative associations among the variables. In particular, although CS was specified before NE, the reverse ordering cannot be ruled out. Future research incorporating longitudinal data or cross-lagged designs may help clarify the directionality and temporal ordering of the observed relationships.

Second, because all variables were derived from the same reporting source, the findings may be affected by shared measurement limitations. Neither Harman’s single-factor test nor the CLF analysis pointed to major common method bias. Still, same-source, same-time self-report data mean that common method variance remains a possibility. In addition, self-report measures may be subject to response biases, including self-report bias and social desirability bias, as participants may unintentionally or intentionally provide responses that differ from their actual experiences. Future research could employ a multi-source design and include objective indicators to obtain a more comprehensive assessment of the study variables.

Third, convenience sampling was used, with participants coming from just one university in Hainan Province. This feature may restrict how far the results can extend to EFL learners in other settings, regions, or institutions. Future research could recruit more diverse samples from multiple regions, schools, and cultural backgrounds to examine the robustness of the observed associations.

Finally, although CS and NE were found to be associated with the relationship between PPs and SA, the present study did not include all potentially relevant individual and contextual factors. Unmeasured variables, such as personality characteristics, learning motivation, family support, or educational environment, may represent potential sources of residual confounding. Later work could explore additional individual and environmental elements possibly pertinent to SA in EFL populations.

## 7 Conclusion

Drawing on the PERMA model, this study examined the associations among PPs, CS, NE, and SA among EFL learners. The findings indicated a positive association between PPs and SA. Significant indirect associations were observed through CS and NE separately, and a significant sequential indirect association was also observed through CS and NE, consistent with the hypothesized sequential indirect-association model. Among the indirect pathways, the association involving NE accounted for the largest proportion of the total association, highlighting the relevance of emotional experiences in understanding SA among EFL learners. Overall, these findings provide a more differentiated understanding of the psychological factors associated with SA. However, given the cross-sectional design and the use of convenience sampling from a single institution, these findings should not be interpreted as establishing causal or temporal relationships, and their generalizability to the broader EFL learner population remains limited.

## Data Availability

The raw data supporting the conclusions of this article will be made available by the authors, without undue reservation.
